# Immune checkpoint inhibitors and renal toxicity

**DOI:** 10.1016/j.heliyon.2024.e31911

**Published:** 2024-05-24

**Authors:** F. Bocchi, S. Häfliger, S. Schmid, D. Sidler

**Affiliations:** a Department for Nephrology and Hypertension, Inselspital, Bern University Hospital, University of Bern, Bern, Switzerland; b Department of Medical Oncology, Inselspital, Bern University Hospital, University of Bern, Bern, Switzerland

**Keywords:** Acute kidney injury, Immune checkpoint inhibitors, Renal transplant, Rechallenging

## Abstract

Immune checkpoint inhibitors (ICIs) have transformed the management of a broad spectrum of cancers as metastatic melanoma, non-small lung cancer or renal cell carcinoma. These humanized monoclonal antibodies target immune-regulatory receptors expressed on T lymphocytes, antigen presenting cells and tumor cells and induce an immunological anti-tumor response. If on the one hand these molecules have led to considerable improvement in survival outcomes, on the other hand these therapies can be associated with immune-related adverse effects (irAEs). While these side effects have become well known, the best diagnostic and therapeutic approaches are still under investigation. The authors discuss pathophysiology, clinical presentation and histological features of ICIs renal toxicity. Furthermore, we focus on kidney transplant (KT) recipients, including the therapeutic adaptation approach to immunosuppression and the risk of rejection.

## 1 Introduction

Immune checkpoint inhibitors (ICIs), a type of immunotherapy, have profoundly transformed the world of oncology, becoming nowadays standard of care in the management of several advanced cancers (in particular, melanoma, non-small lung cancer, urothelial and renal cell carcinoma). Expanded prescription of immunotherapy has resulted in a significant improvement in overall outcomes and patient survival, even in patients with metastatic cancer [1]. On the other side, these drugs have side effects and are associated with uncontrolled stimulation of the immune system resulting in an increasing risk of developing immune-related adverse effects (irAEs) of different magnitude in any organ system. The skin, the gastro-intestinal tract, liver and the endocrine system are most often involved with incidence ranging between 15 and 90 % [1]. Renal injury is reported less frequently and is often underestimated; ranging from 1 to 2% with monotherapy to 4.9 % with combined immunotherapy [[2], [3], [4]]. Kidney damage may be accompanied by other irAEs, particularly skin reactions, which can even occur beforehand. This relationship can be significant for clinicians in tracking and handling irAEs [5,6]. The outcomes can be deleterious, potentially leading to permanent damage of kidney function, which might affect the ability to undergo further antineoplastic therapies. Moreover, a trend for an increased risk of death has been observed in patients with acute kidney injury (AKI) and no full recovery [7]. For this reason, caution by prescribing ICIs, early recognition of renal injury and close collaboration between nephrologist and oncologist is essential in order to find the best therapeutic strategy and to provide quality care to patients.

## 2 Nephrotoxicity of immune checkpoint inhibitors

This widely prescribed class of drugs interacts with receptors or ligands blocking pathways called immune checkpoints resulting in deregulation of immune system. ICIs can suppress T cell activity or promoting self-tolerance through inhibition of cytotoxic T-lymphocyte antigen 4 (CTLA4; ipilimumab, tremelimumab), programmed cell death 1 (PD1; nivolumab, pembrolizumab, cemiplimab) or programmed cell death ligand (PDL1; atezolizumab, avelumab, durvalumab) respectively. ICIs, mainly cleared by proteolytic degradation in the liver, have extended half-lives, spanning from 6 to 27 days, with variations depending on the specific ICI agent [8]. Renal dose adaptation is not necessary, and the use of ICIs up to advanced stages of renal impairment is not contraindicated [9]. Stimulation of the immune system by these drugs can results in irAEs, with some organ systems being more affected than others. All of the different ICI classes can be involved, with anti-CTLA4 and combinations bringing major risk [10]. Older age, lower baseline eGFR (estimated glomerular filtration rate), hypertension, prior or concomitant irAEs, pre-existing genitourinary malignancy, the use of proton pump inhibitor (PPI) and others nephrotoxic medications (antibiotics, non-steroidal anti-inflammatory treatment, cisplatin/carboplatin) were independently associated with AKI and could contribute to specific auto-immune complications in patients treated with ICIs [3,7].

### 2.1 Patterns of kidney injury

Knowledge of kidney pattern injury has evolved over time and various types of lesions have been identified, of which AKI remains the most common, typically caused by acute interstitial nephritis (AIN). Other nephropathies such acute tubular necrosis, glomerulonephritis and acute thrombotic microangiopathy have also been described [11]. Moreover, ICIs have been associated with an increased risk of rejection in kidney transplant (KT) patients. The mean time from exposure to ICIs to the onset of renal damage is highly variable, ranging from 6 to 37 weeks [11] with a median time of 14–16 weeks [3,10]. The presence of irAEs in other organs increases suspicion but is not a sensitive indicator of ICI-induced AKI [6]. In fact, according to a multicenter study, 43 % of patients with ICI-induced AKI, had also a concurrent irAEs [12]. The pathophysiology of renal injury is not fully elucidated. While a link exists between ICIs use and the onset of AIN, the underlying mechanism promoting glomerular damage are less well understood and have to be better elucidated.

### 2.2 Acute interstitial nephritis

AIN is the most common pattern of injury detected in >90 % of patients who underwent a renal biopsy [1,8,11,13]). Sterile pyuria, leucocytes casts and hematuria are often accompanying signs with poor specificity and sensitivity. The clinical behavior differs from the classical AIN induced by others medications. Biopsy usually shows edema, interstitial inflammation and lymphocytic infiltrates, with varying degrees of plasma cells and eosinophils. In a few cases, granulomas can also be present [13]. Renal damage can occur at any time, according to some studies approximately 3–16 months after drug exposure [2]. The molecular mechanisms underlying AIN associated with ICI involve intricate interactions within the immune system and renal tissue. While exact pathways remain unclear, several hypotheses have been proposed. ICIs may activate auto-reactive T cells, initiating an immune attack on renal tubular cells and leading to AIN. Additionally, ICI therapy can disrupt immune checkpoints, resulting in excessive T cell activation and cytokine release, which contributes to kidney inflammation and damage. Some individuals may have a predisposition to drug hypersensitivity reactions, including AIN, triggered by ICI therapy. Furthermore, variations in immune-related genes can influence susceptibility to AIN following ICI therapy, affecting immune responses and contributing to the development of AIN. Finally, environmental factors like concurrent medications or infections may interact with ICI therapy, exacerbating immune-mediated kidney injury and promoting AIN development [14]. Indeed, concomitant use of non-steroidal anti-inflammatory drugs, PPIs or antibiotics have been identified as risk factors [2,8].

### 2.3 Glomerular disease

Glomerular diseases related to ICIs are less common and have been reported sporadically. They may manifest as a nephrotic or nephritic syndrome. Pauci-immune glomerulonephritis, podocytopathy (including minimal change disease and focal segmental glomeruloslerosis) and C3 glomerulopathy are the most frequently described glomerular disorders with a reported frequency of 27 %, 20 % and 11 % respectively [1,4]. Aqeel et al. suggested that ICIs, specifically PD-1 inhibitors, could cause de novo anti-neutrophil cytoplasmic antibody (ANCA) associated vasculitis or trigger a relapse [15]. A systemic review summarized cases with positive anti-myeloperoxidase (MPO) ANCA serology and in most cases related to the administration of nivolumab, followed by pembrolizumab [4]. Others case reports have described positive anti-proteinase-3 (PR-3) or negative ANCA serology in relation to the use of ipilimumab or atezolizumab use [16]. In contrast to ANCA associated vasculitis where dialysis is often necessary, podocytopathies present with relatively well maintained kidney function and nephrotic proteinuria. Other glomerular disorders, such as immunoglobulin A nephropathy, anti-glomerular membrane disease, lupus-like nephritis and membranous nephropathy have also been described [13]. It is worth noting that AIN has been commonly observed in up to 40 % of cases involving glomerular lesions [8]. The postulated pathophysiological mechanism is characterized by a broad stimulation of the immune system's self-reactivity, lymphocyte infiltration in the renal interstice, deposition of immune complexes, microangiopathic endothelial damage, release of cytokines, and tissue injury, ultimately leading to renal dysfunction [17].

## 3 Management

**Fig. 1:**
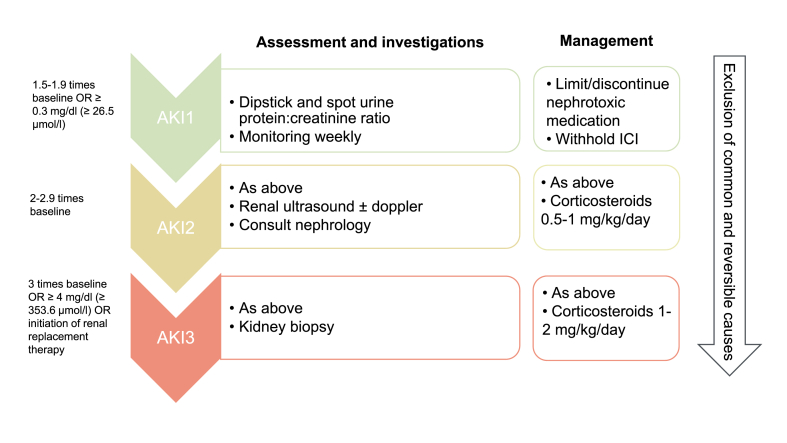
Assessment, investigations and recommended management of acute kidney injury in the context of treatment with immune checkpoint inhibitors. Adapted according to ASCO and NCCN clinical practice guidelines. AKI: Acute Kidney Injury, ICI: Immune checkpoint inhibitor.

AKI in cancer patients is a frequent entity and has extensively studied [18,19]. Effective management depends on its early recognition. A detailed history and clinical examination is essential to rule out others common and reversible causes of AKI, to avoid discontinuation of potentially effective therapies and costly and invasive investigations. No clinical or laboratory data can be used to differentiate with certainty the type of renal lesion, which is why a renal biopsy is often necessary. Due to lack of studies, there is few available data regarding the exact moment of referral to a nephrologist or to introduce a treatment. This explains that only a small proportion of patients have a diagnosis of ICIs nephrotoxicity confirmed by a nephrologist [20]. Recommendations are based on published daily clinical practice and guidelines [18]. In general, several authors advise monitoring patients with mild cases of AKI (creatinine value of 1-1.5x baseline or proteinuria < 1g/day), when glomerular disease is not suspected, and referring the others for a specialist consultation and consideration for kidney biopsy [1,2]. Choosing which patients should undergo a biopsy is one of the most intricate and subjectively determined choices for a nephrologist. The result is of paramount importance, from a prognostic point of view but also by facilitating the appropriate therapeutic choice. Based on published guidelines (National Comprehensive Cancer Network [19]; American Society of Oncology [18]), a kidney biopsy is often recommended but frequently postponed, and patients are more commonly treated empirically (Fig. 1).

Initial treatment is mainly based on corticosteroids 0.5–1 mg/kg/day (preceded, in severe cases by intravenous pulse-dose corticosteroids for 3 days). No tapering therapy as been prospectively evaluated and treatment may need to be continued for longer periods (>4 weeks to 3 months). Gupta et al. showed that early treatment with corticoids (within 14 days following AKI) is associated with higher odds of renal recovery [3]. Concomitant medications that may promote AKI should be discontinued, including considering withholding ICI for grade 2 nephrotoxicity (creatinine 2-3x above baseline) and permanently discontinuation of treatment for grade 3 or 4 (creatinine > 3x baseline) [10,18]. If there is no response to corticosteroids in the first few weeks, the addition of another immunosuppressive therapy (mycophenolate mofetil, azathioprine, cyclosporine, infliximab, cyclophosphamid or rituximab) should be reassessed. Overall, the oncological prognosis of the patient should be considered and the therapy must be individualized [10,13]. Response to treatment is variable. In most of cases, the AIN is responsive to steroid with an 85 % response rate to achieve partial or completely recovery [8,12]. In fewer than 10 % of instances, there is a permanent loss of kidney function and dialysis is required. This could potentially impact the patient's suitability for other anticancer treatments. Glomerular disease and simultaneous extra-renal irAEs were associated with a poor outcome [4,11]. Additional research is needed to establish optimal therapies for addressing these less common kidney complications.

### 3.1 Rechallenging

Re-exposure to immunotherapy after renal injury remains a subject of great debate and relies on a thorough evaluation of patient- and cancer-related factors, taking into account the risk-benefit ratio. Very limited data are available. In some cases, the use of ICIs remains the best therapeutic strategy to treat cancer and maintaining its remission. Thus rechallenging should be considered in any patient, particularly if no others therapeutics options are available. The median waiting time to rechallenge after withdrawal is unknown. The degree of AKI, the existence of a glomerular disease, its recovery as well as the severity of extra-renal irAEs should be considered before a rechallenge. Patients requiring significant recovery time >6 weeks are at higher risk of developing permanent renal damage [10]. In a multicenter study, Cortazar et al. reported a recurrence of AKI in 23 % of 138 patients with short latency period between rechallenging and AKI-episode (in most cases, rechallenge with the same ICI) [12]. Similarly, Gupta et al. described AKI recurrence with a median time of 10 weeks after rechallenge in 20 of 121 patients (16.5 %), of which 40 % developed severe AKI. After ICI therapy interruption and corticosteroid administration, renal recovery was observed in 60 % of cases [3]. An individualized approach is advised, and teamwork between oncologists and nephrologists is essential in establishing the best treatment strategy. Restarting immunotherapy once AKI has resolved with a low corticosteroid intake could potentially prevent further kidney damage; however, this approach has not yet been studied and lacks proven benefits [2]. Additionally, changing cancer drug target and/or deescalating from combined-to mono-therapy may be a safe end effective approach for ICI-rechallenge. Close and regular monitoring (including creatinine, urine and electrolytes) of these patients is essential.

## 4 ICIs and kidney transplant

KT patients have a very high risk of developing cancer (estimated at 3-4x higher than the general population). It is the 2nd cause of death in this group of patients [1]. There is little literature and significant amount of uncertainty concerning the use of ICI and their benefit in KT patients as the latter have been excluded from clinical trials, which is why prescribing ICIs can represent a challenge. Indeed, the activation and proliferation of T cells elicit a robust immune response, increasing the risk of acute rejection (cellular most often observed than humoral). On the other side, the use of immunosuppression can compromise and minimize the antitumor activity of immunotherapy. In a recently published multicenter retrospective cohort study of 69 kT patients with advanced cutaneous squamous cell carcinoma or melanoma receiving ICIs, 42 % developed acute rejection (50 % mixed acute and antibody-mediated rejection, 50 % pure T cell-mediated rejection), 65 % of whom lost their allograft and required dialysis. Median time from ICIs initiation to graft rejection was 24 days [21]. This relatively rapid onset contrasts with the delayed onset of others ICI-AKI in native kidney described above. Murakami et al. also demonstrated that deceased-donor KT status and a higher number of immunosuppression therapies (triple as opposed to dual immunosuppression) at the time of ICI initiation are associated with a lower risk of graft rejection [21]. In a systematic review by Fisher et al. it was noted that the timeframe between KT and the start of ICI treatment is not linked to whether patients encountered rejection or not. Moreover, graft rejection was not the most frequent cause of death in this population of patients, the latter being linked to metastatic disease progression [22]. With respect to immunosuppression, there are no guidelines on the best approach. The conversion of calcineurin inhibitors to mTOR (mammalian target of rapamycin) inhibitors before ICIs initiation is an interesting and emerging therapeutic strategy. This class of drug, with intrinsic antitumor properties, can reduce the development of malignancies [9]. Furthermore, mTOR inhibitors contribute to graft tolerance and have been associated with a better and longer rejection-free graft survival and overall graft survival during concomitant ICI treatment [21]. Finally, anti-CTLA4 molecules seem to be safer compared to anti-PD-1/PD-L1 therapies and are associated with a lower risk of graft rejection [1]. Although systematic data on the choice and dose of immunosuppression are lacking, individual adaptation and tailoring of immunosuppression is the cornerstone of the therapy in KT patients with malignancies. This purpose requires a close monitoring, collaboration with a multidisciplinary approach.

## 5 Conclusion and future directions

Cancer patients who develop AKI require special attention in order to make an early diagnosis and administer appropriate therapy. Currently, noninvasive predictive biomarkers (blood, urine, DNA and gene expression) and imaging-based biomarkers are being studied in order to identify early kidney lesions linked to ICI. These advancements hold the potential to enhance clinical diagnosis, therapy, and prognosis for this patient population [10]. A biopsy is not always necessary. However, given the broad spectrum of renal impairment and the lack of other non-invasive diagnostic tools, this should be considered with no delay if there is no improvement in kidney function after correction of reversible causes and suspension of ICIs. Treatment with corticosteroids is recommended, particularly in cases of confirmed AIN and a rechallenge should be discussed in any patients once the initial injury has been resolved. Although the ICI-AKI prognosis is often good with a recovery in most of the case, clinicians should remain vigilant and caution is advised in prescribing ICIs. With the increasing prescription of ICIs, further larger studies are needed to better understanding the incidence, risk factors and outcomes as well as in-depth studies of others new high specific tools facilitating diagnosis and recently marketed drugs belonging to this class.

## Data availability statement

No data was used for the research described in the article. No data associated with the study has been deposited into a publicly available repository.

## CRediT authorship contribution statement

**F. Bocchi:** Writing – review & editing, Writing – original draft. **S. Häfliger:** Writing – original draft. **S. Schmid:** Writing – original draft. **D. Sidler:** Writing – review & editing, Writing – original draft.

## Declaration of competing interest

On behalf of all authors (Simon Häfliger, Sabine Schmid, Daniel Sidler), i declare that the article has been written in the absence of any commercial or financial relationships that could be construed as a potential conflict of interest.
